# Effects of Dietary Inclusion of *Ocimum gratissimum* and *Vernonia amygdalina* Leaf Meals on Growth Performance, Carcass Traits, Blood Profile, and Gastrointestinal Parasites in Weaner Rabbits

**DOI:** 10.1155/vmi/1803252

**Published:** 2026-02-12

**Authors:** Basile Konmy, Christian C. Dansou, Fiacre L. M. Acakpo Doumavo, Fallone B. Ganyé, Tony T. B. A. Sounkere, Rodrigue Towanou, Erick Virgile Bertrand Azando, Lamine Baba-Moussa, Sanny-Yo Doko Allou, A. Pascal Olounladé

**Affiliations:** ^1^ Zootechnical Research and Livestock System Unit, School of Management and Operation of Livestock Systems, Doctoral School of Animal and Fisheries Sciences, National University of Agriculture, Ketou, Benin; ^2^ Laboratory of Biology and Molecular Typing in Microbiology, Faculty of Sciences and Techniques, University of Abomey-Calavi, Abomey-Calavi, Benin, uac.bj; ^3^ Laboratory of Ecology, Health and Animal Production, Faculty of Agronomy, University of Parakou, Parakou, Benin, univ-parakou.com

**Keywords:** antiparasitic activity, feed efficiency, *Ocimum gratissimum*, rabbit, *Vernonia amygdalina*

## Abstract

This study evaluated the effect of *Ocimum gratissimum* and *Vernonia amygdalina* supplementation on the growth performance, feed intake, blood profile, excretion of helminth eggs, and coccidial oocysts in growing rabbits through two experiments. Eighty‐four New Zealand White rabbits aged 40–50 days and averaging 790.04 ± 60.70 g, divided into 7 treatments of 12, were used in the first experiment for 56 days. Ninety rabbits aged 40–45 days and weighing 600 ± 50 g, divided into 10 treatments of 9 young rabbits, were used in the second experiment for 28 days. OG and VA leaves were harvested, dried, milled, and incorporated into the diet at 0% (control), 5%, 10%, and 15%. The treatments in Experiment 1 included a control treatment, as well as OG and VA administered at inclusion levels of 5%, 10%, and 15% each. Measured parameters included feed intake, feed conversion ratio, blood hematological and biochemical indices, and carcass characteristics. The treatments in Experiment 2 comprised OG−, OG supplemented at 5%, 10%, and 15%, and OG+ and VA−, VA supplemented at 5%, 10%, and 15%, and VA+’. Parameters measured included fecal excretion of oocytes and helminth eggs. A one‐factor analysis of variance followed by linear and quadratic regression was performed on the production data, and then a generalized linear model was carried out on the egg and oocyst excretion data using the statistical software R. Significance was considered at *p* < 0.05. The results demonstrated that dietary inclusion of OG and VA leaf meals significantly improved feed intake, feed conversion ratio, average weight gain, and carcass yield (*p* < 0.05) compared to the control. In contrast, the excretion of helminth eggs and fecal coccidial oocysts was significantly reduced (*p* < 0.001), with an improvement in hematocrit levels (*p* < 0.05) at 15% supplementation. These findings suggest that incorporating OG and VA leaves at 15% in rabbit diets can enhance growth performance and effectively reduce gastrointestinal parasite loads.

## 1. Introduction

Rabbits are commercially raised worldwide, providing meat and economic benefits to low‐ and middle‐income countries due to their efficient conversion of feed into valuable products. They are prized for their lean protein and have a rapid growth rate and short reproduction cycle [1, 2]. With 70.9% of global production, Asia is the world’s leading producer of rabbit meat, followed by Europe, Africa, and America [3]. Despite rabbits’ ability to make efficient use of fiber‐rich forages such as grasses and legumes, their productivity in developing countries is hampered by problems such as feed shortages and insufficient availability of high‐quality feed that adequately supports the nutritional requirements of the animals. These constraints are further exacerbated by inadequate technical support and insufficient knowledge of rabbit farming practices among smallholders, restricting the possible gains from rabbit farming in terms of improved food security and livelihoods [4]. To address the issues related to feed shortages, incorporating unconventional local leaf supplements into rabbit diets offers a promising solution [5, 6]. Several tropical leaf meals have shown promising effects on rabbit productivity. Studies have indicated their positive influence on feed intake (FI) and nutrient digestibility [7–9], as well as improvements in weight gain, feed conversion ratio (FCR), and carcass yield [10]. Additionally, these leaf meals have been associated with reduced feed costs [10–12]. Several tropical leaf meals have shown promising effects on rabbit productivity. Inclusion of *Moringa oleifera* at 10%–30% improves carcass yield, live weight, litter size, and economic returns without adverse effects [13]. *Indigofera zollingeriana* up to 12% supports nutrient utilization and growth with no negative impact [14].

Among alternative leaf supplements, *Ocimum gratissimum* (OG) and *Vernonia amygdalina* (VA) have attracted attention for their potential health benefits in both humans and animals. OG is particularly promising as a nonconventional feed material, frequently used in Africa and Asia for culinary and medicinal purposes [15]. Belonging to the Lamiaceae family with therapeutic properties, aromatic OG is native to Africa but is widespread in Asia and Latin America. It is utilized in zootechnics due to its antimicrobial and antioxidant effects mainly attributed to its richness in volatile compounds such as eugenol, thymol, and carvacrol. Several studies have demonstrated that its inclusion in poultry feed improves digestibility, reduces intestinal microbial load, and promotes gut health, while effectively replacing growth‐promoting antibiotics [16, 17]. Numerous studies have explored the effects of supplements and feed additives on animal health [16], with OG leaf extracts often being used in broiler chickens to reduce microorganism loads and enhance growth performance. However, there is limited information relative to the medicinal and nutritional effects of supplementing the OG leaf meal in rabbits. VA, known as “bitter leaf,” is part of the Asteraceae family and is recognized for its diverse biological activities, such as antiparasitic, antihyperglycemic, antioxidant, antiplasmodial, anti‐inflammatory, and antibacterial properties [18, 19]. Native to tropical Africa, it is widespread in West and Central Africa, notably Nigeria, Benin, and Cameroon. The plant is traditionally used for its antiparasitic, antioxidant, and immunostimulant properties. In animal nutrition, VA is increasingly appreciated as a phytogenic additive, notably in poultry and small ruminant feeds. Studies have shown that its incorporation into the diet improves growth, feed conversion, and pathogen resistance, thanks to secondary metabolites such as flavonoids, saponins, alkaloids, and tannins [20, 21]. Its leaves are rich in secondary metabolites such as tannins, flavonoids, phenolic compounds, quinones, and alkaloids [22–24]. The impact of fresh leaves of OG and VA and of aqueous and acetone extracts of VA leaf powder on oocysts and helminth eggs was evaluated in rabbits [25, 26]. In rabbits, the antimicrobial properties of OG and VA have been studied, but the zootechnical and antiparasitic performances are poorly documented. Consequently, the supplementation rates of these plants have clearly not been studied in rabbits.

Although the effect of OG and VA on zootechnical performance in poultry and small ruminants has already been explored [27], this study stands out for its focus on these parameters in rabbits. It uniquely evaluates the impact of these two plants on growth performance, carcass traits, and the reduction in gastrointestinal parasitism, a combination of parameters that remains largely unexplored in this species. This study offers a significant advance by combining the assessment of growth performance, carcass traits, and gastrointestinal parasite control, while also exploring the optimal dietary levels of OG and VA in rabbits—an area that remains insufficiently characterized in current research. This research contributes valuable knowledge regarding the practical integration of OG and VA leaves into rabbit diets as a means of mitigating feed constraints in low‐resource contexts. The study aimed to investigate the dose‐dependent impact of these botanical supplements on growth performance, carcass characteristics (Experiment 1), and gastrointestinal parasitism (Experiment 2) in rabbits. The hypotheses tested were that diets containing OG and VA improved performance and reduced parasites, and their effects were dose‐dependent.

## 2. Materials and Methods

### 2.1. Study Area

The study was conducted at the Livestock Facility of the Faculty of Agricultural Sciences’ experimental farm, University of Abomey‐Calavi (UAC), located in the Atlantic Department in southern Benin. The facility provides controlled conditions suitable for experimental studies on animals, particularly for nutritional and health‐related research. The climatic conditions of the study area are subequatorial, featuring two distinct rainy seasons and two dry periods annually, with an average temperature near 27°C and relative humidity varying between 65% and 95% [28].

### 2.2. Animal Ethics Statement

This research adhered to the ethical guidelines established by the Declaration of Helsinki. All animal experiments followed ARRIVE guidelines, and protocols involving experimental rabbits were reviewed and approved by the animal welfare committee of the National University of Agriculture in Porto‐Novo, Benin (Approval Number: 143‐2018/President‐CER/SA).

### 2.3. Processing of VA and OG Leaves and Diet Formulation

Seven experimental diets were formulated, consisting of a control diet and six other diets supplemented with VA or OG leaf meals at inclusion levels of 5%, 10%, and 15%. Fresh leaves of OG and VA (Supporting Figure S1) were harvested from mature plants (10–12 weeks old) cultivated at the experimental farm of the Faculty of Agricultural Sciences, UAC (Benin). After harvesting, the leaves were washed with clean water, shade‐dried at ambient temperatures (26°C–30°C) for seven days, and ground into a fine powder using a locally fabricated hammer mill equipped with a 1‐mm sieve. The ingredients were individually weighed, milled, and thoroughly blended using a vertical feed mixer. The complete mixtures were pelletized using a flat‐die pelletizer with a 4 mm die diameter (KL120B, China‐made model), operating at ambient temperature without the addition of steam or binding agents, to preserve the integrity of the phytogenic compounds. Diet formulation followed the nutritional recommendations for growing rabbits as described by Kpodekon et al. [29] and Drogul et al. [30]. A total of 50 kg of each diet was prepared in a single batch, stored in a cool, dry, and ventilated environment, and used throughout the experimental period (Table 1).

**TABLE 1:** Composition of experimental diets with different levels of inclusion of OG and VA leaf meals (%).

Ingredients (%)	Control	OG5	OG10	OG15	VA5	VA10	VA15
Maize	14	14	14	14	14	14	14
Rice bran	20	20	20	20	20	20	20
Cottonseed meal	7	7	7	7	7	7	7
Soybean meal	4	4	4	4	4	4	4
Corn bran	14	14	14	14	14	14	14
Palm kernel cake	40	40	40	40	40	40	40
Lysine	0.1	0.1	0.1	0.1	0.1	0.1	0.1
Methionine	0.1	0.1	0.1	0.1	0.1	0.1	0.1
Oyster shell	0.6	0.6	0.6	0.6	0.6	0.6	0.6
Dicalcium phosphate	0.2	0.2	0.2	0.2	0.2	0.2	0.2
*O. gratissimum* leaf meal	—	5	10	15	—	—	—
*V. amygdalina* leaf meal	—	—	—	—	5	10	15
Total	100	105	110	115	105	110	115

*Note: O. gratissimum* and *V. amygdalina* leaf meals have been added to the feed diet as dietary supplements. Control = control diet; OG5: 5% *O. gratissimum* leaf meal; OG10: 10% *O. gratissimum* leaf meal; OG15: 15% *O. gratissimum* leaf meal; VA5: 5% *V. amygdalina* leaf meal; VA10: 10% *V. amygdalina* leaf meal; VA15: 15% *V. amygdalina* leaf meal.

### 2.4. Chemical Analysis of Experimental Diets

Chemical analyses of the formulated diets and the OG and VA leaf meals were carried out at the Laboratory of Food and Animal Nutrition (LANA) of the Faculty of Agricultural Sciences, UAC (Benin). The analyses included the determination of dry matter (DM), organic matter (OM), crude protein (CP), fat (F), crude fiber (CF), neutral detergent fiber (NDF), non‐nitrogenous extracts (NNEs), and mineral content. DM and ash (mineral matter) contents were measured according to methods established by the French Standards Association [31]. F content was determined by the Soxhlet extraction method and CP content by the Kjeldahl method (N∗6.25) [31]. CF content was analyzed following the Weende method, whereas NDF content was determined according to the method described by Van Soest and Wine [32]. The metabolizable energy (ME) of the leaf meals and experimental diets was estimated using the following regression equation:

ME (kcal/kg) = 3951 + 54.4 × MG − 40.8 × MM − 88.7 × CF, where MG is the percentage of F, MM is the percentage of mineral matter, and CF is the percentage of CF. This equation was previously validated for feedstuffs used in rabbit nutrition [33].

### 2.5. Animals and Management

In the first experiment, 84 New Zealand rabbits of both sexes, aged 40–50 days at weaning and weighing 773.585 ± 57.8 g, were used. For the second experiment, 90 New Zealand rabbits of both sexes, aged 40–45 days at weaning and weighing 600 ± 50 g, were used. The rabbits were placed in cages built from steel, each with dimensions of 80 cm (length), 45 cm (width), and 35 cm (height). Feed and water were provided manually to the rabbits. Feed was distributed once daily into stone troughs, and clean drinking water was also supplied *ad libitum* using the same type of stone troughs placed in the cages. A 14‐day adaptation phase was observed to allow the rabbits to become accustomed to their new living environment.

### 2.6. Experimental Design

In the first experiment, 84 rabbits were assigned to seven treatments with four replicates. Each replicate was made up of three rabbits. Each treatment was fed a treatment diet, corresponding to the following dietary treatments: control, VA5%, VA10%, and VA15%, and OG5%, OG10%, and OG15%. A daily diet of 300 g was distributed in each cage as follows: 150 g in the morning at 7 am and 150 g in the evening at 4 pm. Feed refusals were weighed. Daily dietary allocations were adjusted when necessary to avoid wastage. For the duration of the experiment, the animals had unlimited access to water. The experiment lasted 56 days.

In the second experiment, 90 rabbits were assigned to 10 treatments with three replicates. Each replicate was made up of three rabbits. These treatments were respectfully marked as follows: O−, O+, OG5%, OG10%, OG15%, V−, V+, VA5%, VA10%, and VA15%. The O− and V− treatments served as negative controls, whereas the O+ and V+ treatments served as positive controls. Positive control treatment was treated with sulfadimethoxine (at a dose of 0.5 g per liter in drinking water for 5 days) and piperazine (at a dose of 300 mg/kg, in drinking water for 5 days) according to the manufacturer’s instructions, whereas negative control groups received no treatment. Test treatments OG5%, OG10%, and OG15% were fed diets containing 5%, 10%, and 15% OG leaf meal, respectively, whereas test treatments VA5%, VA10%, and VA15% were fed diets containing 5%, 10%, and 15% VA leaf meal, respectively. Throughout the 28‐day experiment, coprological analyses were carried out to calculate helminth egg and oocyst excretion rates.

### 2.7. Natural Infestation

To assess the dose effect of OG and VA leaf meals on fecal excretion in the second experiment, initial qualitative coproscopic examinations were conducted during the selection phase, before the experiment, to ensure that all rabbits included in the trial exhibited a detectable but comparable level of parasitic infection. Parasitized rabbits were then allocated to the different treatment groups in such a way as to maintain a similar infestation level across groups, thereby establishing a homogeneous baseline. Subsequently, during the experimental period, fecal samples were collected from each treatment group at various time points to monitor the evolution of parasite excretion under the influence of the dietary treatments. On the scheduled fecal collection days, fine‐mesh nets (approximately 1 mm^2^ mesh size, similar in appearance to standard mosquito netting) were installed early in the morning, around 6:00 a.m., under each cage. This timing allowed for the collection of fresh fecal pellets before the production and ingestion of cecotrophs by the rabbits. The nets retained the newly excreted feces while permitting dust and fine particles to fall through. Collected feces were harvested a few hours later for coproscopic analyses. After collection, the nets were carefully cleaned and removed until the next scheduled sampling. The number of eggs and oocysts per gram of feces was quantified by analyzing freshly collected samples in the laboratory using the standard McMaster method [34]. Representative images of helminth eggs and oocysts are shown in Supporting Figure S2. To determine eggs per gram of feces (EPG), the McMaster technique employs the following formula:

(1) EPG or OPG=N∗FV∗M,

where EPG (or OPG) represents the number of EPG (or the number of oocysts per gram of feces), *N* is the total number of eggs observed in both grids of the McMaster counting chamber, *F* is the dilution factor of the sample (the ratio of sample volume to flotation liquid volume used), *V* represents the total volume of the sample analyzed, typically measured in milliliters (mL), and *M* is the weight of the fecal matter used to prepare the sample (usually in grams).

This formula estimates the number of parasite eggs present in 1 g of feces based on the number of eggs observed in a sample examined.

### 2.8. Production Performance Index

Throughout the experimental period, several zootechnical parameters were measured to comprehensively assess the impact of dietary treatments on rabbit productivity. These included average weight gain (AWG), average FI (AFI), FCR, and carcass yield. These indicators are widely recognized as reliable measures of growth efficiency, feed utilization, and slaughter performance in rabbit production systems. The parameters were determined based on the following equations.

The AWG is determined as follows:

(2) AWG g/period=Weight of the previous week−average weight of the current week growth period .

AFI is calculated as follows:

(3) AFI g=Quantity of distributed feed−quantity of remaining feed Period given d.

FCR is calculated as follows:

(4) FCR=Average feed intake g/dayAverage weight gained g/day.

The carcass yield is calculated as follows:

(5) Carcass yield %=Carcass weight gLive weight at slaughter g×100,

which has been determined according to the previous study [35].

### 2.9. Hematological and Biochemical Parameters

Hematological and serum biochemical indices were assessed to evaluate the effects of the dietary treatments on blood indices. Blood samples (*n* = 9 per group) were collected from the marginal ear vein using sterile syringes. For each rabbit, 3 mL of blood was drawn: One portion was placed into ethylenediaminetetraacetic acid (EDTA) tubes for hematological analysis and another into dry tubes for serum biochemical analysis.

Hematological parameters measured included white blood cell (WBC) count, red blood cell (RBC) count, hemoglobin (Hb) concentration, and platelet count. Serum biochemical analyses focused on liver function indicators, including alanine aminotransferase (ALT), aspartate aminotransferase (AST), gamma‐glutamyl transferase (GGT), alkaline phosphatase (ALP), and serum total protein (TP). All analyses were carried out at a biomedical analysis laboratory, following established protocols for hematological and biochemical analysis.

### 2.10. Calculation of Treatment Efficacy

The antiparasitic efficacy of dietary supplementation was assessed using the fecal egg count (FEC) reduction test (FECRT), following the guidelines recommended by the World Association for the Advancement of Veterinary Parasitology (WAAVP). Efficacy was calculated as the percentage reduction in FECs or oocyst counts between pretreatment (Day 0) and post‐treatment (Day 14 or Day 28). Two FECRT percent efficacy estimates were calculated using the following formula, as described in previous studies by McKenna [36]:

(6) FECRT1100 %=1−T2T1,

(7) FECRT2100 %=1−T2C1,

where *T*1 is the mean pretreatment fecal nematode egg and coccidial oocyst counts of a treated group, *T*2 is the mean post‐treatment fecal nematode egg and coccidial oocyst counts of a treated group, and *C*1 is the mean pretreatment fecal nematode egg and coccidial oocyst counts of an untreated control group.

### 2.11. Statistical Analyses

Differences between groups were assessed using a one‐way ANOVA followed by Duncan’s post hoc test. The *p*‐value was calculated using Student’s *t*‐test. The normality of the data was tested using the Shapiro–Wilk test, and the relationships between the different parameters were highlighted using the Bravais–Pearson correlation coefficient. Polynomial contrasts were used to examine linear and quadratic responses to increasing levels of OG and VA leaves in the diets. Quadratic regressions (*y* = ax^2^ + bx + *c*) were fitted to the responses of the dependent variables to dietary OG and VA leaves. The response to the extremum for the OG and VA leaves was determined as Cp = −*b*/(2∗*a*). The results are reported as means ± standard error of the mean (SEM). Statistical analyses were performed using R version 4.2.3, and significance was set at *p* < 0.05. Graphical representations were generated using GraphPad Prism 9.00.

## 3. Results

### 3.1. Chemical Analysis of Experimental Diets Supplemented With OG and VA Leaf Meals

The increasing incorporation of OG and VA in diets has shown interesting effects on the nutritional quality of the diets. There was a progressive increase in DM, CP, and digestible and ME contents, suggesting a nutritional densification of feeds (Table 2). VA, particularly at 15%, stood out with the highest concentrations of digestible protein (23.54%) and energy (3156.20 kcal/kg), indicating a high potential as a natural protein and energy supplement. In addition, OG significantly enriched diets in total fiber (NDF and ADF), cellulose (up to 10.75%), and water‐insoluble pectins (WIPs), which could have a positive effect on intestinal transit, microbiota health, and digestive regularity in rabbits. These improvements in fibrous fractions, particularly insoluble ones, are essential in rabbit diets to prevent digestive disorders.

**TABLE 2:** Proximate composition and detergent fiber fractions of experimental diets.

Item	Control	OG5	OG10	OG15	VA5	VA10	VA15
Dry matter (%)	89.10	93.60	95.10	97.60	93.60	95.10	97.60
Crude ash (%)	4.40	4.76	5.12	5.48	4.73	5.07	5.40
Crude protein (%)	26.79	27.24	27.69	28.14	27.75	28.72	29.68
Fat (%)	2.07	2.12	2.17	2.22	2.29	2.50	2.72
Crude fiber (%)	8.04	8.94	9.84	10.74	8.43	8.82	9.21
NDF (%)	18.52	20.66	22.80	24.94	20.08	21.64	23.20
ADF (%)	10.02	11.08	12.14	13.20	10.49	10.96	11.43
ADL (%)	2.18	2.27	2.36	2.45	2.24	2.30	2.36
WIP (insoluble pectin) (%)	4.07	5.32	6.57	7.82	4.32	4.57	4.82
Starch (%)	24.65	24.65	24.65	24.65	25.55	26.45	27.35
Total sugars (%)	5.04	5.34	5.64	5.94	5.14	5.24	5.34
Digestible protein (%)	21.51	21.73	21.96	22.18	22.18	22.86	23.54
Digestible energy (kcal/kg)	3008.03	3132.03	3256.03	3380.03	3144.53	3281.03	3417.53
Metabolizable energy (kcal/kg)	2775.20	2896.20	3017.20	3138.20	2902.20	3029.20	3156.20

*Note:* OG5: 5% *O. gratissimum* leaf meal; OG10: 10% *O. gratissimum* leaf meal; OG15: 15% *O. gratissimum* leaf meal; VA5: 5% *V. amygdalina* leaf meal; VA10: 10% *V. amygdalina* leaf meal; VA15: 15% *V. amygdalina* leaf meal; NDF: neutral detergent fiber; ADF: acid detergent fiber; ADL: acid detergent lignin.

The essential amino acid profile was improved by enriching the diet with VA and OG, albeit only moderately. Lysine, methionine, threonine, and sulfur amino acid contents all showed a progressive increase, particularly marked with treatment VA15% as shown in Table 3. In terms of minerals, OG was distinguished by a significant increase in calcium, magnesium, and potassium levels, reaching higher levels than the control (Table 3).

**TABLE 3:** Amino acids and mineral profile of experimental diets.

Item	Control	OG5	OG10	OG15	VA5	VA10	VA15
Lysine (%)	1.45	1.47	1.50	1.53	1.48	1.51	1.53
Methionine (%)	0.43	0.44	0.45	0.46	0.44	0.46	0.48
Total sulfur amino acids (%)	0.90	0.91	0.93	0.94	0.93	0.97	1.00
Threonine (%)	1.01	1.03	1.05	1.08	1.04	1.08	1.11
Tryptophan (%)	0.35	0.36	0.36	0.37	0.36	0.36	0.37
Calcium (%)	0.19	0.23	0.27	0.31	0.20	0.21	0.22
Phosphorus (%)	0.54	0.55	0.55	0.56	0.59	0.63	0.67
Sodium (%)	0.02	0.03	0.04	0.05	0.03	0.04	0.05
Chlorine (%)	0.08	0.08	0.09	0.09	0.09	0.10	0.11
Magnesium (%)	0.24	0.25	0.26	0.27	0.25	0.27	0.29
Potassium (%)	1.15	1.17	1.19	1.22	1.19	1.24	1.29

*Note:* OG5: 5% *O. gratissimum* leaf meal; OG10: 10% *O. gratissimum* leaf meal; OG15: 15% *O. gratissimum* leaf meal; VA5: 5% *V. amygdalina* leaf meal; VA10: 10% *V. amygdalina* leaf meal; VA15: 15% *V. amygdalina* leaf meal.

### 3.2. FI and Growth Performance

Effects exerted by the inclusion of VA and OG leaf meals on FI and growth performance of the rabbits are presented in Table 4. At 28 days, the VA10% (1419.33 g) and VA15% (1417.83 g) diets achieved the highest mean weights, whereas the control group remained the lightest (1260.83 g), with a marginal linear trend (*p* = 0.055). At 56 days, although the OG10% treatment achieved the highest weight (2083.83 g), closely followed by VA15% (2051.83 g), no significant difference was found (*p* = 0.385). In terms of AWG, the VA15% diet produced the best performance between Days 1 and 28 (660.50 g), followed by OG10% (616.00 g), whereas the control group showed the lowest gain (487.25 g). This difference was significant (*p* = 0.031). During the second phase (Days 28–56), the best gain was recorded in the control animals (710.42 g), whereas OG5% showed the lowest gain (574.00 g), with no statistically significant difference. Over the whole period (Days 1–56), VA15% (1294.50 g), OG10% (1289.17 g), and OG15% (1283.17 g) showed the highest cumulative gains, whereas OG5% (1066.17 g) showed the lowest performance (*p* = 0.092). FI was found to be very significantly influenced by diet (*p* < 0.001). Between Days 1 and 28, OG15% (1662.06 g) and VA15% (1505.17 g) stimulated consumption, whereas OG5% (1106.83 g) recorded the lowest intake. This trend continued over the following period (Days 28–56), with OG15% (2082.39 g) and VA15% (1897.61 g) being the highest values, compared with OG5% (1267.56 g). Over the 56 days, OG15% (3744.44 g) had the highest intake, followed by VA15% (3402.78 g), whereas OG5% (2374.39 g) remained the lowest. VA15% (2.58) and OG10% (2.82) revealed the lowest consumption index, and OG5% (4.03) revealed the highest index. From Days 28 to 56, OG10% was the most effective (2.20), whereas VA10% showed a very high index (5.45). Over the entire study period (1–56 days), OG10 and VA15 were the most effective (2.51 and 2.87, respectively), whereas VA10 (4.48) was the least effective (Table 4).

**TABLE 4:** Effects of dietary inclusion of OG and VA leaf meals on growth performance of growing rabbits.

	Control	OG5	OG10	OG15	VA5	VA10	VA15	SEM	A	L	Q	Quadratic equation	*R* ^2^	Peak
AW,g												—	—	—
IW (D1)	773.58	770.17	794.67	775.83	810.33	848.33	757.33	60.70	0.953	0.928	0.710	—	—	—
D28	1260.83	1262.33	1410.67	1366.33	1319.67	1419.33	1417.83	71.21	0.472	0.055	0.137	—	—	—
FW (D56)	1971.25	1836.33	2083.83	2059.00	2001.67	2032.67	2051.83	75.59	0.385	0.125	0.302	—	—	—
AWG, g														
D28	487.25^b^	492.17^b^	616.00^a^	590.50^a^	509.33^b^	571.00^a^	660.50^a^	40.59	0.031	0.001	0.004	*Y* = 0.118*X* ^2^ + 8.775*X* + 473.917	0.249	−37,088
D56	710.42	574.00	673.17	692.67	682.00	613.33	634.00	37.78	0.209	0.716	0.307	—	—	—
D1–D56	1197.67	1066.17	1289.17	1283.17	1191.33	1184.33	1294.50	56.63	0.092	0.034	0.055	—	—	—
FI, g														
D28	1231.69^d^	1106.83^e^	1455.61^c^	1662.06^a^	1235.17^d^	1523.28^b^	1505.17^b^	5.19	< 0.001	< 0.001	< 0.001	*Y* = 1.2613*X* ^2^ + 10.289*X* + 1174.226	0.755	−4079
D56	1693.67^d^	1267.56^f^	1423.00^e^	2082.39^a^	1831.28^c^	1883.28^b^	1897.61^b^	8.49	< 0.001	0.001	1.859	—	—	—
D1–D56	2925.36^d^	2374.39^e^	2878.61^d^	3744.44^a^	3066.44^c^	3406.56^b^	3402.78^b^	9.84	< 0.001	< 0.001	< 0.001	*Y* = 6.065*X* ^2^ − 41.875*X* ^2^ + 2866.480	0.6052	3452
FCR														
D28	3.18	4.03	2.82	3.91	3.39	3.52	2.58	0.64	0.774	0.763	0.906	—	—	—
D56	2.50	2.33	2.20	3.19	3.27	5.45	3.17	0.51	0.196	0.417	0.556	—	—	—
D1–D56	2.84	3.18	2.51	3.55	3.33	4.48	2.87	0.47	0.336	0.654	0.684	—	—	—

*Note:* SEM: standard error of the means (*n* = 12; individual rabbits per diet); A: *p*‐value of GLM; L: *p*‐value of linear effect of dietary inclusion levels; Q: *p*‐value of quadratic effect on dietary inclusion levels; d: days of age. OG5: 5% *O. gratissimum* leaf meal; OG10: 10% *O. gratissimum* leaf meal; OG15: 15% *O. gratissimum* leaf meal; VA5: 5% *V. amygdalina* leaf meal; VA10: 10% *V. amygdalina* leaf meal; VA15: 15% *V. amygdalina* leaf meal.

Abbreviations: AW, average weight; AWG, average weight gain; FCR, feed conversion ratio; FI, feed intake.

^a,b,c,d,e^Means followed by different letters in the same row differ significantly at the 5% level (*p* < 0.05).

Quadratic equation analysis reveals a differentiated influence of leaf supplementation on growth performance, feed consumption, and carcass traits of the rabbits. Weight gain at 28 days is influenced by supplementation, but the coefficient of determination (*R* ^2^ = 0.25) indicated that this parameter remains moderately predictable from the variable tested. The peak of the curve is estimated at negative supplementation (−37.08%), which is biologically unrealistic, suggesting instead an increasing linear response in the interval studied (0%–15%). In contrast, FI was well explained by the models, particularly at 28 days (*R* ^2^ = 0.76), with an estimated peak at −4.08%, indicating a progressive increase in intake with supplementation over the entire range studied. For the total period (Days 1–56, *R* ^2^ = 0.61), the curve shows a minimum around 3.45%, reflecting an increase in FI (Table 4).

Quadratic effects of the different levels of OG and VA supplementation (0%–15%) on growth performance, feed consumption, and carcass characteristics in rabbits are shown in Figure 1. In general, the curves show that the animals’ response to supplementation is not linear, but varies according to the variables considered, with different optimum points. Weight gain at 28 days (Figure 1(a)) increases steadily with supplementation, indicating an increasing positive effect up to 15%. In contrast, FI at Day 28 and over the whole period (Figures 1(b) and 1(c)) follows a U shape, suggesting that consumption is lower at intermediate levels of supplementation (around 5%) and increases thereafter. This may indicate better feed efficiency at moderate doses, before potential overconsumption at high levels.

FIGURE 1Quadratic modeling of the effects of supplementation of OG and VA leaf meals on the zootechnical parameters of rabbits. (a) Weight gain at 28 days, (b) feed intake at 28 days, (c) cumulative feed intake (Days 1–56), (d) abdominal fat mass, (e) spleen weight, (f) carcass yield, and (g) kidney weight. Each curve is the result of a quadratic regression fit, whose equation is displayed on the corresponding graph. These curves visualize optimal responses or inflection thresholds as a function of supplementation level.

**Figure:**
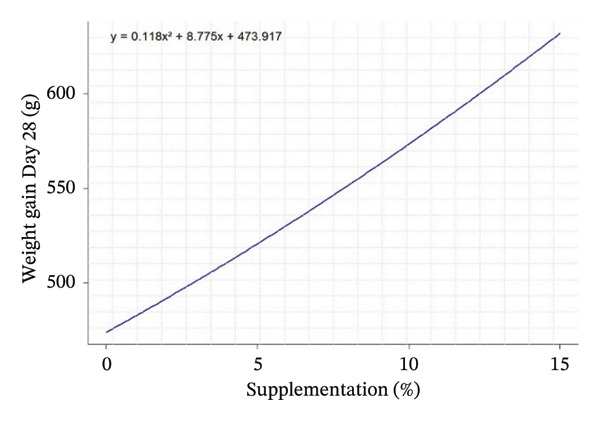
(a)

**Figure:**
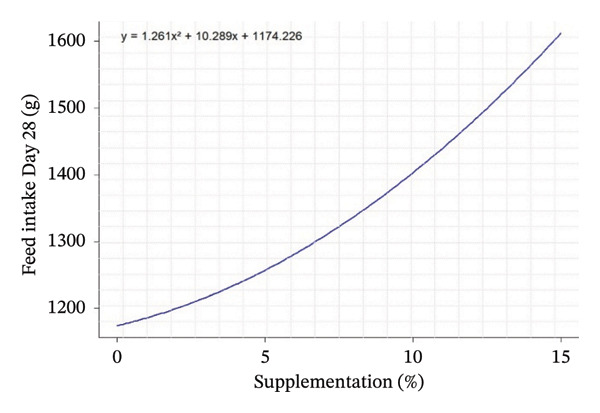
(b)

**Figure:**
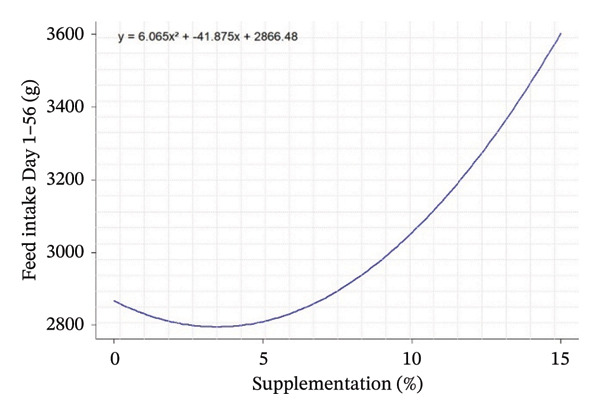
(c)

**Figure:**
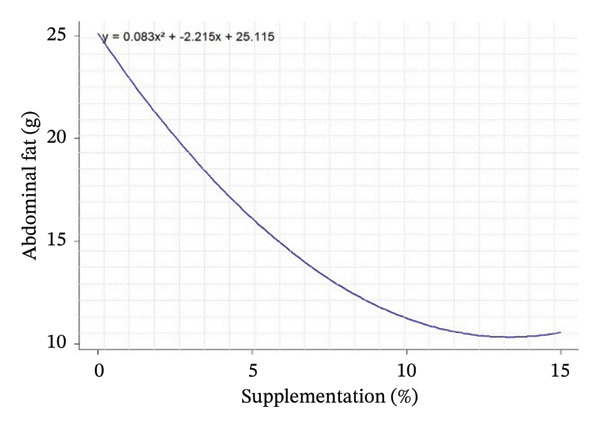
(d)

**Figure:**
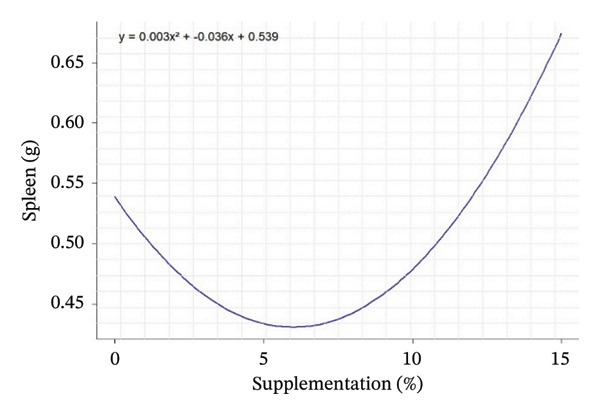
(e)

**Figure:**
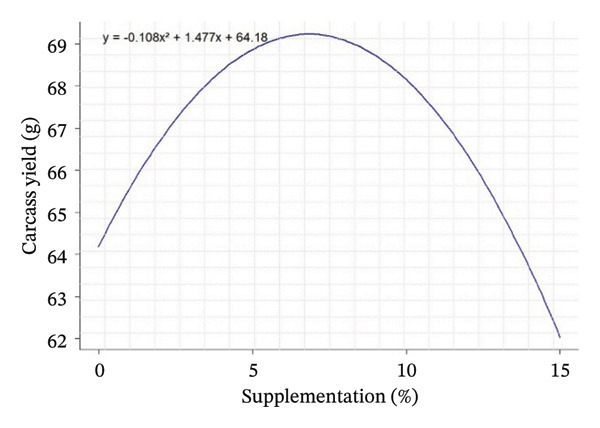
(f)

**Figure:**
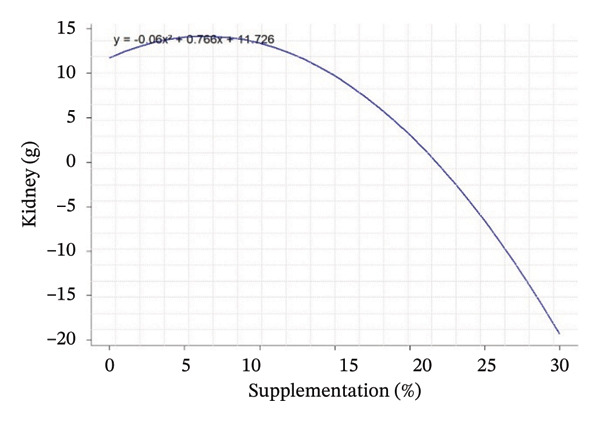
(g)

### 3.3. Effects of the Inclusion of VA and OG Leaf Meals on the Carcass Characteristics of Rabbits

Effects of the inclusion of VA and OG leaf meals on carcass characteristics are shown in Table 5. The inclusion of OG and VA leaf meals significantly improved the carcass yield of rabbits. Carcass yield in the study showed a slight increase in groups OG5%, OG10%, and VA5%, followed by a reduction at higher doses in groups OG15% and VA15%. The control group had a yield of 64.81%, whereas the other groups ranged from 60.79% to 69.60%, with a significant effect for groups OG5% and VA10%. The highest carcass yields were achieved with the OG10% diet, followed by VA5% and VA10%, respectively (*p* < 0.001). Moreover, there were significant differences in the weights of organs such as the heart, liver, and kidneys among the tested groups (*p* < 0.05). Kidney weight exhibited an increase, with both linear (*p* = 0.002) and quadratic (*p* < 0.001) responses being significant. Conversely, abdominal F decreased in the tested lots, with both linear and quadratic responses being significant (*p* < 0.001). Additionally, the spleen displayed significant variation according to both linear and quadratic responses (*p* < 0.001) (Table 5).

**TABLE 5:** Effects of the inclusion of VA and OG leaf meals on the carcass characteristics of rabbits.

	Control	OG5	OG10	OG15	VA5	VA10	VA15	SEM	A	L	Q	Quadratic equation	*R* ^2^	Peak
Carcass yield (%)	64.81^bc^	67.25^ab^	68.66^ab^	60.79^c^	68.62^ab^	69.60^a^	62.84^bc^	1.38	0.001	0.054	< 0.001	*Y* = 0.083*X* ^2^ − 2.215X + 25.115	0.648	13,374
Head (g)	116.33	131.48	116.36	108.37	122.32	124.55	119.56	5.04	0.099	0.220	0.080			
Heart (g)	4.94	4.42	4.61	4.71	6.61	6.11	6.83	0.22	0.060	0.203	0.444			
Liver (g)	45.01^b^	40.88^b^	54.15a	47.12^ab^	44.08^b^	42.74^b^	42.13^b^	1.91	0.001	0.509	0.698			
Kidney (g)	11.53^bc^	13.26^ab^	12.67bc	10.37^bd^	15.43^a^	13.43^a^	9.11^d^	0.52	< 0.001	0.002	< 0.001	*Y* = −0.06*X* ^2^ + 0.766*X* + 11.726	0.617	6346
Abdominal fat (g)	25.19^a^	12.15^c^	8.06^d^	14.13^c^	19.84^b^	14.65^c^	6.83^d^	0.60	< 0.001	< 0.001	< 0.001	*Y* = 0.083*X* ^2^ − 2.215*X* + 25.115	0.648	13,374
Spleen (g)	0.55^c^	0.42^e^	0.52^d^	0.72^b^	0.43^e^	0.54^d^	0.76^a^	0.02	< 0.001	< 0.001	< 0.001	*Y* = 0.003*X* ^2^ − 0.036*X* + 0.539	0.809	5416

*Note:* SEM: standard error of the means (*n* = 12; individual rabbits per diet); A: *p*‐value of GLM; L: *p*‐value of linear effect of dietary inclusion levels; Q: *p*‐value of quadratic effect on dietary inclusion levels; d: days of age. OG5: 5% *O. gratissimum* leaf meal; OG10: 10% *O. gratissimum* leaf meal; OG15: 15% *O. gratissimum* leaf meal; VA5: 5% *V. amygdalina* leaf meal; VA10: 10% *V. amygdalina* leaf meal; VA15: 15% *V. amygdalina* leaf meal.

^a,b,c,d,e^Means followed by different letters in the same row differ significantly at the 5% level (*p* < 0.05).

Carcass traits such as abdominal F mass (*R* ^2^ = 0.65; peak 13.37%), spleen weight (*R* ^2^ = 0.81; peak 5.42%), carcass yield (*R* ^2^ = 0.42; peak 6.87%), and kidney weight (*R* ^2^ = 0.62; peak 6.35%) also show sufficiently high *R* ^2^ values to indicate that supplementation has a structured and measurable effect on these indicators. Thus, supplementation influences not only FI but also body composition in rabbits, with levels of explained variability that support the biological relevance of the models obtained (Table 5).

Regarding the evolution of quadratic curves for carcass characteristics, abdominal F (Figure 1(d)) shows an inverted U‐shaped curve, suggesting that intermediate levels of supplementation limit F deposition. Spleen weight (Figure 1(e)) shows a U‐shaped curve, reflecting a possible sensitivity of the immune system to supplementation. Carcass yield (Figure 1(f)) follows a bell‐shaped curve, peaking at around 7% supplementation, which appears to be the optimum level for maximizing feed‐to‐meat conversion. Kidney weight (Figure 1(g)) decreases after an initial peak, indicating possible physiological adaptation or metabolic overload at high doses. Quantile–quantile (Q–Q) plots and residuals versus fitted value plots were used to visually evaluate the distributional characteristics of zootechnical parameters; the plots are shown in Supporting Figure S3.

### 3.4. Effects of the Inclusion of OG and VA Leaf Meals on Hematological and Serum Biochemical Parameters of Rabbits

Hematological parameters showed no significant variation (*p* > 0.05) between parameters. However, the VA15% group had the highest number of WBCs, whereas RBCs were more numerous in the OG5% group (Table 6).

**TABLE 6:** Hematological parameters of rabbits fed diets supplemented with varying levels of OG and VA leaf meals.

	Control	OG5	OG10	OG15	VA5	VA10	VA15	SEM	*p*‐Value
WBC	6.732	7.618	6.67	7.2	7.812	7.438	8.766	0.70	0.469
Neutro	34.2	42.4	40.2	35.8	42.6	37	34.8	2.21	0.053
Eosino	2.6^ab^	2.8^ab^	2.2^b^	3^a^	2.2^b^	2.2^b^	2.4^b^	0.18	0.038
Lympho	59.4	51.2	53.8	57.4	51.8	56.8	58.8	2.12	0.062
Mono	3.8	3.6	3.8	3.8	3.4	4	4	0.34	0.928
Hb	10.44	12.26	12.12	10.84	11.46	11.1	11.58	0.84	0.741
RBC	4.562	5.062	4.928	4.562	5.014	4.718	4.654	0.23	0.572
PCV	31.32	37.74	37.18	32.88	33.74	32.52	34.48	2.53	0.55
BP	165.8	153.4	154.8	175.6	147	167.8	177.2	8.27	0.196

*Note:* WBC = white blood cell (10^3^/L); Neutro = neutrophils (%); Eosino = eosinophils (%); Lympho = lymphocytes (%); Mono = monocytes (%); Hb = hemoglobin (g/dL); RBC = red blood cell (10^6^/mm); PVC = packed volume cell (%); BP = blood platelets (10^3^/mm). OG5: 5% *O. gratissimum* leaf meal; OG10: 10% *O. gratissimum* leaf meal; OG15: 15% *O. gratissimum* leaf meal; VA5: 5% *V. amygdalina* leaf meal; VA10: 10% *V. amygdalina* leaf meal; VA15: 15% *V. amygdalina* leaf meal. SEM: standard error of the means.

^a,b^Means followed by different letters in the same row differ significantly at the 5% level (*p* < 0.05).

Hematocrit levels fluctuated throughout the experiment as shown in Figure 2. For subjects receiving VA leaf meal, hematocrit levels ranged from 28% to 30%, whereas it was 29% in the control group (Figure 2(a)). Subjects receiving OG leaf meal exhibited hematocrit levels ranging between 28% and 31%, which was 33% higher than that of the control group (Figure 2(b)). By Day 28, hematocrit levels were significantly improved (*p* < 0.05) in the various groups that received leaf meal from both plants compared to their respective controls. Eosinophil values ranged from 2.2% to 3.0% depending on diet, with the OG15% group having the highest value (3.0), whereas several groups, including OG10%, VA5%, and VA10%, had significantly lower values (2.2) (*p* = 0.038).

FIGURE 2Comparative hematocrit values of rabbits under different dietary treatments. (a) Hematocrit values of rabbits fed with OG leaf meal. (b) Hematocrit level of rabbits fed with VA leaf meal; OG5: 5% *O. gratissimum* leaf meal; OG10: 10% *O. gratissimum* leaf meal; OG15: 15% *O. gratissimum* leaf meal; VA5: 5% *V. amygdalina* leaf meal; VA10: 10% *V. amygdalina* leaf meal; VA15: 15% *V. amygdalina* leaf meal. ns: not significant (*p* > 0.05);  ^∗^ *p* < 0.05;  ^∗∗^ *p* < 0.01; (Dunnett’s test). Multiple comparisons were made against negative controls.

**Figure:**
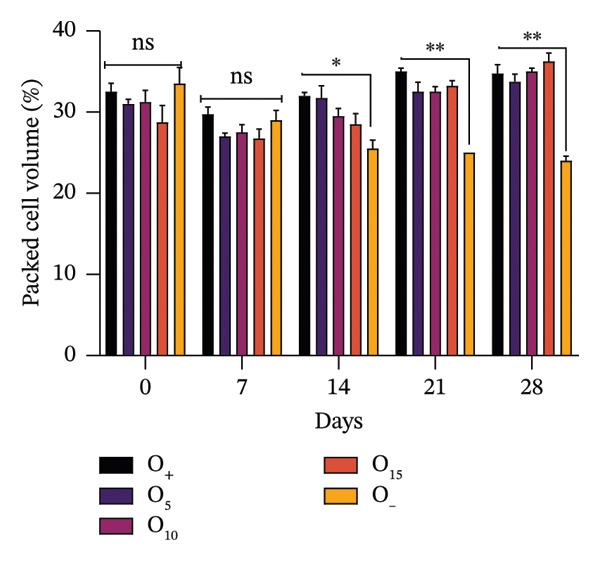
(a)

**Figure:**
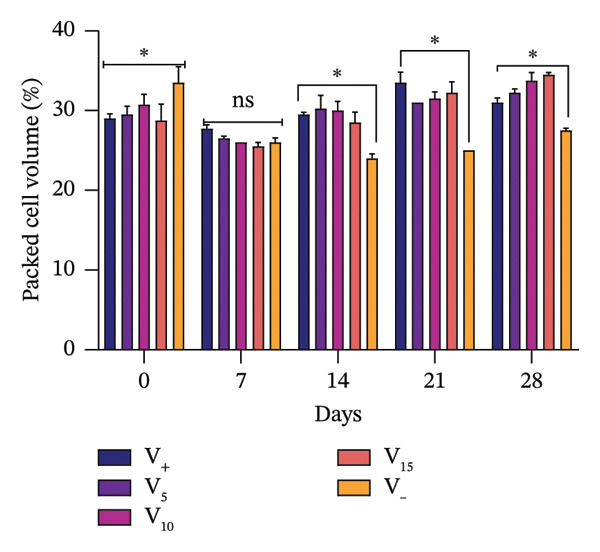
(b)

Urea and creatinine values representing renal parameters did not differ (*p* > 0.05). Liver parameters marked by AST, alanine aminotransferase (ALT), and alkaline aminotransferase (ALP) showed nonsignificant variation (*p* > 0.05). Treatment VA15% had the lowest urea concentration, and VA5% had the lowest creatinine concentration (*p* > 0.05). The control treatment had the lowest AST value, and the VA10% treatment had the lowest ALT value (Table 7).

**TABLE 7:** Biochemical parameters of rabbits fed diets supplemented with varying levels of OG and VA leaf meals.

	Control	OG5	OG10	OG15	VA5	VA10	VA15	SEM	*p*‐Value
Urea	0.29	0.36	0.35	0.29	0.37	0.30	0.28	0.03	0.112
Crea	15.17	14.84	15.31	12.42	11.60	12.18	12.84	1.11	0.215
AST	26.26	83.38	57.13	90.67	49.12	55.9	64.59	20.62	0.523
ALT	91.10	85.60	87.81	80.41	80.81	76.58	83.57	5.11	0.559
ALP	150.8	173.6	176.8	124	172.6	120.4	123.2	19.49	0.372
TP	62.4	72.6	67.2	67.8	70	63.2	69.6	6.92	0.946

*Note:* Urea = serum urea (g/L); Crea = serum creatinine (g/L); ALT = alanine aminotransferase (IU); AST = aspartate aminotransferase (IU); ALP = alkaline phosphatase (IU); TP = total protein (g/L); OG5: 5% *O. gratissimum* leaf meal; OG10: 10% *O. gratissimum* leaf meal; OG15: 15% *O. gratissimum* leaf meal; VA5: 5% *V. amygdalina* leaf meal; VA10: 10% *V. amygdalina* leaf meal; VA15: 15% *V. amygdalina* leaf meal. SEM: standard error of the means.

### 3.5. Variation in Coccidial Oocyst and Helminth Egg Counts Following Dietary Inclusion of OG and VA Leaf Meals

Effects of variation in coccidial oocyst and helminth egg counts following dietary inclusion of VA and OG leaf meals are presented in Figure 3. In rabbits receiving different doses of VA, oocyst excretion decreased from Day 7^e^ in all treated groups. This reduction became more marked the higher the dose, with a particularly marked drop in the VA15% treatment from Day 14^e^. At 28 days, the VA10% and VA15% treatments reach excretion levels similar to those of the positive control. In contrast, the negative control treatment maintained a stable, high oocyst load throughout experimentation (Figure 3(a)). With regard to helminth egg excretion, a progressive reduction was also observed in the treatment group with VA leaves, particularly for VA10% and VA15%. These two treatments show a marked drop in EPG from Day 7^e^, reaching low levels at 28 days. Treatment VA5% performs less well, whereas the untreated treatment maintained relatively constant parasite loads (Figure 3(b)). The effect of OG leaves on oocyst excretion follows a trend comparable to that observed with VA. Treatments OG10% and OG15% show a significant drop in OPG from Day 7^e^, with minimum values reached at D28. The OG15% treatment demonstrates efficacy close to the positive control, whereas the OG treatment maintained a high load (Figure 3(c)). Finally, EPG analysis in rabbits receiving OG also revealed a progressive reduction in helminth eggs over time. The deworming effect is particularly marked in the OG10% and OG15% treatments, where a rapid and sustained reduction is observed up to 28 days. Conversely, animals in the OG treatment show a tendency toward increased excretion (Figure 3(d)).

FIGURE 3
*In vivo* effect of VA leaf meal on the excretion of several oocysts and helminth eggs from naturally infested rabbits. (a) VA leaf meal on the number of oocysts; (b) VA leaf meal on helminth egg excretion; (c) OG leaf meal on the number of oocysts; (d) OG leaf meal on helminth egg excretion. OG5: 5% *O. gratissimum* leaf meal; OG10: 10% *O. gratissimum* leaf meal; OG15: 15% *O. gratissimum* leaf meal; VA5: 5% *V. amygdalina* leaf meal; VA10: 10% *V. amygdalina* leaf meal; VA15: 15% *V. amygdalina* leaf meal.

**Figure:**
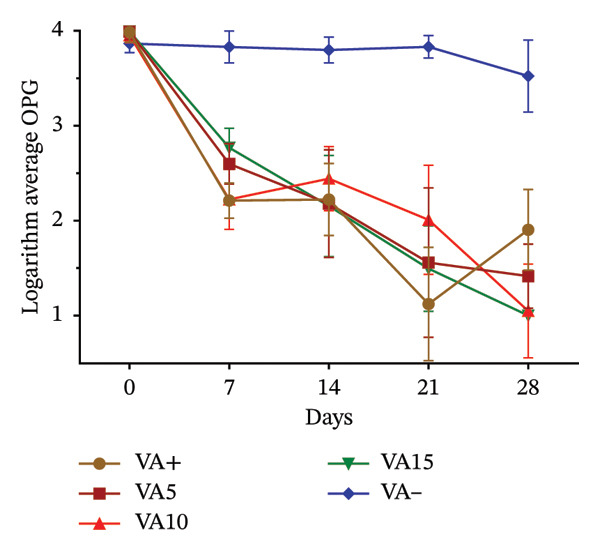
(a)

**Figure:**
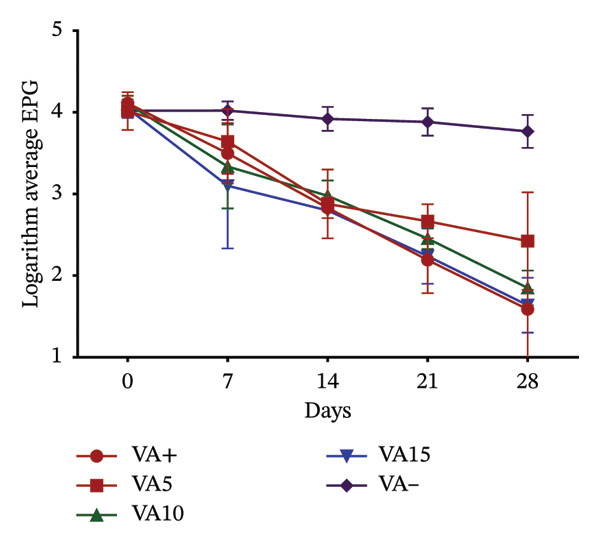
(b)

**Figure:**
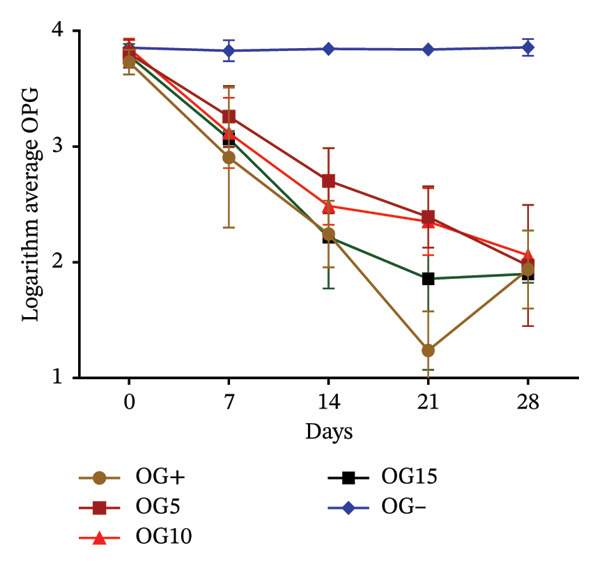
(c)

**Figure:**
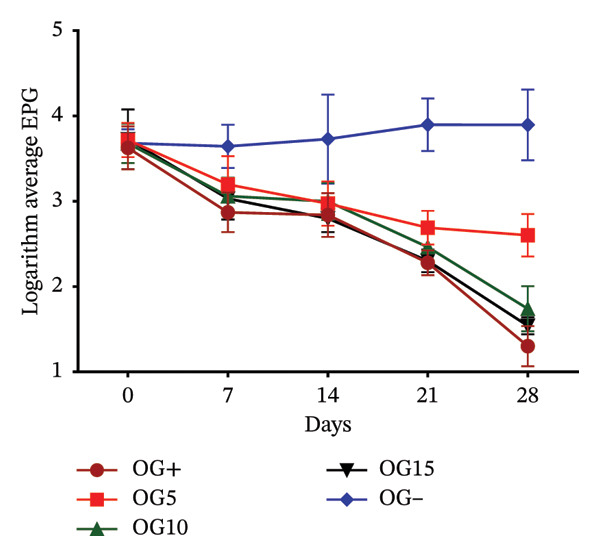
(d)

### 3.6. Efficacy of OG and VA Leaf Meals in Reducing Oocyst and Helminth Egg Counts in Rabbits

Evaluation of the antiparasitic efficacy of OG and VA leaves, measured by the FECRT, revealed overall high antiparasitic activity against oocysts and moderately variable activity against helminths. In the case of oocysts, FECRT values were high from D14 and were maintained at D28, often exceeding 90%, whatever the treatment. Notably, doses of 10% VA and 15% OG showed sustained efficacy (≥ 94%) up to D28. However, no statistically significant differences were detected between treatments (*p* > 0.05), suggesting that both plants have a comparable effect at all doses tested against oocysts (Table 8).

**TABLE 8:** *In vivo* antiparasitic efficacy of OG and VA leaf meals on the excretion of coccidial oocysts and helminth eggs from naturally infested rabbits.

Parasites	Treatments	FECRT1		FECRT2		SEM	Significance
		D14	D28	D14	D28		
Oocysts	OG5	71.65	92.29	69.27	92.35	5.21	ns
	OG10	78.99	94.06	79.23	94.53	4.36	ns
	OG15	83.26	94.73	80.96	95.60	3.87	ns
	VA5	94.02	91.58	95.76	91.78	9.88	ns
	VA10	97.29	94.02	97.9	94.46	4.49	ns
	VA15	91.17	96.89	93.78	97.99	4.20	ns

Helminths	OG5	85.21	88.97	69.86	82.01	4.14	ns
	OG10	89.20	88.32	75.92	89.16	3.25	ns
	OG15	89.91	93.61	79.81	95.17	3.45	ns
	VA5	49.86^b^	88.97^a^	51.67^b^	87.49^a^	10.83	s
	VA10	72.11	91.32	76.91	92.26	5.09	ns
	VA15	77.65	96.61	78.92	97.42	5.42	ns

*Note:* OG5: 5% *O. gratissimum* leaf meal; OG10: 10% *O. gratissimum* leaf meal; OG15: 15% *O. gratissimum* leaf meal; VA5: 5% *V. amygdalina* leaf meal; VA10: 10% *V. amygdalina* leaf meal; VA15: 15% *V. amygdalina* leaf meal; FECRT: fecal egg count reduction test. ns: no significance (*p* > 0.05); s: significance (*p* < 0.05). Values followed by different letters (a, b) in the same row differ significantly at the 5% level (*p* < 0.05).

In contrast, efficacy against helminths was more heterogeneous. FECRT ranged from 49.86% (VA5%) to 89.91% (OG15%) at D14. At D28, a general improvement was observed, reaching up to 97.42% for VA15%. The only statistically significant difference (*p* < 0.05) was observed for treatment VA5% between Days 14 and 28, where efficacy increased from 49.86% to 88.97% (FECRT1) and from 51.67% to 87.49% (FECRT2). This significant improvement reflects a cumulative effect over time, particularly marked at low doses for VA. For the other treatments, although the trends were similar, the differences were not statistically significant (Table 8).

## 4. Discussion

The incorporation of OG and VA leaves gradually improved the nutritional value of the experimental diets. Proximate analysis showed a steady increase in DM, protein, ash, and lipids as the inclusion level increased. Fiber fractions (CF, NDF, and ADF) also increased, particularly in diets containing OG. This result is consistent with previous observations that the use of leaves in feed tends to increase the structural fiber content [12, 37]. Conversely, VA contributed more to the intake of starch and soluble sugars, which explains the higher values of digestible and ME, whereas OG increased the proportion of insoluble pectin. These differences reflect qualitative variability between the two plants in terms of the parietal fraction.

The amino acid and mineral profile also confirmed the nutritional value of both leaves. Essential amino acids such as lysine, methionine, threonine, and tryptophan increased gradually with inclusion, with VA appearing to further enrich sulfur‐containing amino acids. This is particularly important because lysine and methionine are often limiting amino acids in rabbit diets [38]. Mineral intake was also enhanced: OG further improved calcium content, whereas VA provided more phosphorus and potassium. These results confirm that tropical leafy vegetables are significant sources of protein and minerals in animal feed [11, 39]. The CP content remained well above the minimum 17%–18% required for growth, and the ME (above 2500 kcal/kg) was sufficient to ensure maintenance and growth [38]. The balance of amino acids and minerals suggests that these diets can effectively support health and zootechnical performance. Furthermore, these results corroborate previous work showing that the incorporation of unconventional forages or phytochemical additives can improve the nutritional value of rabbit diets while reducing dependence on traditional ingredients [37].

The inclusion of OG and VA leaf meals had no negative effect on the growth performance and general health of the rabbits. Rabbits supplemented with 10% and 15% OG and VA, respectively, showed weight gain, an improved feed conversion rate, and a reduction in excretion of coccidial oocysts and helminth eggs. These performances are probably due to the richness of these plants in secondary metabolites with antiparasitic and nutritional effects, such as tannins, flavonoids, and alkaloids. These results surpass those reported by Abdel‐Wareth et al. [40], who used oregano leaf in rabbit feed, suggesting that differences in plant composition may account for the disparity.

Similarly, Chen et al. [35] reported higher AWG with a 10% supplement of *Cajanus cajan* compared to our study. Indeed, *C. cajan* is a legume richer in protein than VA and OG, which could explain the differences observed.

Treatments OG10% and VA15% showed superior growth performance at 28 days, as evidenced by the highest AWGs and improved FCRs compared with the other treatments. In particular, treatments OG10% and VA15% achieved the highest AWGs, and treatment OG10% recorded the highest feed conversion index, indicating improved feed utilization efficiency, particularly at the beginning of the experimental period. The highest AWG and improved FCRs observed in the OG10% and VA15% groups were likely due to the rabbits’ improved physiological assimilation of these diets. However, at 56 days, the differences between the treatments diminished, suggesting that the positive effect of OG and VA on growth may be more marked in the short term but stabilizes in the long term. This could indicate that the effect of these plants on growth is more significant in the early phases of rabbit development. These results are in line with the previous studies [41] carried out on the effect of medicinal plants such as *Syzygium aromaticum* and *Thymus vulgaris* on rabbit breeding. The marked increase in FI observed in treatments receiving higher levels of VA and OG, especially in the OG15% treatment, may be linked to enhanced palatability or appetite‐stimulating effects, potentially attributable to the bioactive properties of OG and VA leaves. These plants have been reported to have nutritional and phytobiotic properties such as mild antimicrobial effects, antioxidant activity or effects, and the property of stimulating enzyme secretion that could encourage FI [42]. Although trends were visible, significant differences were not observed for FCR. This may suggest that the increased FI in some treatments did not translate into improved efficiency in converting feed to body weight.

Our study showed that supplementing with 10% OG and VA leaves improved carcass yield and significantly reduced internal abdominal F. This result is consistent with the work of Sun et al. [13], which shows that *Moringa oleifera* leaves also significantly reduced serum cholesterol levels in rabbits. The improved carcass yield and reduced abdominal F in rabbits fed OG and VA are thought to be mainly linked to their bioactive compounds (secondary metabolites), such as flavonoids, phenolic compounds, and essential oils. Absorption, intestinal health, and lipid metabolism are improved by the secondary metabolites. The result is less F and more lean tissue. These results are similar to those of Apáez‐Barrios et al. [43], who observed improvements in growth performance and carcass traits in fattening rabbits supplemented with 0.5% *Pithecellobium dulce* fruits in feed.

In a study by Kismiati et al. [44], the inclusion of plants such as VA showed positive effects on carcass yield in Japanese quail, although the effects were more marked at low doses of the plant, in line with the results observed here. The increase in carcass yield in the present study can be attributed to the enhanced FI and probably digestion. This effect was not dose‐dependent. Diets based on OG and VA exerted differentiated effects on the internal organs of rabbits, in particular the heart, liver, and kidneys. The modulation of cardiac mass could reflect an adaptive response to the bioactive compounds of VA, without indication of pathophysiological stress, in line with the observations of Tunasamy et al. [45] on its cardioactive effects. The variations observed in the liver could indicate increased metabolic activity or hepatic mobilization in the face of the secondary molecules present, as reported by Linda et al. [46] concerning the hepatoprotective effect of OG. The renal response, marked by a nonlinear evolution, suggests a dose‐dependent sensitivity, in line with the results of Tokofai et al. [21] on the effects of plant extracts on renal function in monogastric animals. These observations underline the importance of optimal dosage to guarantee the beneficial effects of these plants without altering the integrity of the internal organs.

The hematological and biochemical parameters, which are crucial indicators of physiological changes, remained unaffected by the inclusion of VA and OG leaf meals except for eosinophils. All values for the hematological and biochemical indices were within the normal range of values for healthy rabbits reported by Benson and Paul‐Murphy [47]. This suggests that the supplementation, at the doses used, did not have a harmful impact on essential physiological functions, such as immune response, liver or kidney function, or overall blood health. The current results contradict the results of Ezeonu [48] when aqueous extract of VA and OG was supplemented in fattening rabbits. Eosinophils play a key role in immune defense, particularly against helminth parasites and in allergic inflammatory reactions [49, 50]. The significant increase observed with high inclusion of OG suggests an immunomodulatory effect of its secondary metabolites, such as flavonoids and essential oils, which have already been reported to stimulate eosinophil‐mediated responses [51]. This result indicates that OG at high levels of incorporation could enhance immune activation in rabbits. Assessment of the effect of VA leaf meal on rabbits revealed a decrease in the number of RBCs, possibly indicating reduced formation or increased destruction. Conversely, increased WBC counts in treated groups may suggest immune response stimulation or enhanced distribution of blood cells in tissues. Hematocrit levels in rabbits remained relatively constant as reported by El‐Demerdash [52], differing from the findings of Oboh [53], who reported decreased hematocrit due to induced hemolysis in rats treated with aqueous extracts of VA leaf. This disparity may be attributed to differences in animal species and the form of leaf ingestion.

The chosen quantitative coproscopy method represents the primary technique utilized in field surveys, notwithstanding criticisms from authors such as Raynaud et al. [54], who highlighted its general insensitivity and imprecision. Nonetheless, the results obtained in our study indicate that OG and VA possess antiparasitic effects of over 90% against gastrointestinal parasites in rabbits. This efficacy is evidenced by a reduction in parasitic load, with reductions observed in fecal excretion rates of coccidial oocysts from D7 and helminth eggs from D14 for both OG and VA throughout the experiment for FECRT1 and FECRT2 [55, 56]. This effectiveness correlates with an increase in hematocrit rate and weight gain observed in all groups treated with the two plants compared to controls. Previous reports by Danquah et al. [55] have also documented the anthelmintic activity of VA in cattle and against earthworms. In addition, Farombi and Owoeye [57] revealed the anthelmintic effect of VA. The anthelmintic effectiveness of VA in rabbits (99%) exceeds that reported in cattle (59.5%) by Alawa et al. [58]. Notably, our study administered plant leaf meal as part of the rabbit diet, potentially contributing to the higher efficiency observed. Differences in phenological, genetic, and physiological conditions of the plants could also play a role. Our study reported a 96% effectiveness of VA leaf against rabbit coccidial oocysts, a rate lower than the 99.4% reported by Adediran et al. [59] in goats but higher than the 53% reported by Al‐Fifi [60] in free‐range chickens. Variations in species and phenological stage of the plant may explain these differences.

According to the classification of McKenna et al. [61] and the recommendations of the WAAVP, a substance is said to be effective when it reduces the number of eggs/oocysts excreted in the feces by more than 90%. Thus, for all supplementation rates, OG and VA incorporation showed a reduction in helminth eggs/oocysts by over 90%, confirming their efficacy as a natural antiparasitic. The use of OG and VA will provide an inexpensive means of controlling coccidia and helminths in rabbits.

## 5. Limitations

The study’s limitations lie in the absence of techniques such as histopathology and high‐performance liquid chromatography (HPLC), justified by logistical and financial constraints, and an approach based on methods that are accessible and applicable in resource‐poor settings. These methodological choices meet the practical objectives of the study but pave the way for future work incorporating more in‐depth analyses.

## 6. Conclusions

This study demonstrates that dietary supplementation with VA and OG leaves positively influences rabbit production. Notably, these supplements improved growth performance, enhanced carcass characteristics, and reduced gastrointestinal parasitism, without compromising the health status of the animals. Hematological and biochemical analyses confirm their safety and suggest a potential immunomodulatory effect. These findings position OG and VA as promising nonconventional feed resources, particularly valuable in contexts where feed costs and access to conventional antiparasitic treatments are limiting factors. Their use could contribute to more sustainable, cost‐effective, and natural rabbit farming systems, while promoting colony resilience through bioactive plant compounds.

## 7. Recommendations for Further Studies

The absence of improvement in FCR, despite higher intake, raises questions about the digestibility and potential antinutritional factors present in the leaves. Additionally, this study did not explore the interactive effects of OG and VA bioactives, nor their precise mechanisms of antiparasitic action. Meat quality, gut microbiota composition, and long‐term economic outcomes also remain to be investigated. Future research should therefore focus on optimizing inclusion levels, characterizing the plants’ phytochemical profiles, and exploring their impacts on digestibility, microbiota dynamics, and product quality. Such efforts will be essential to validate the practical applicability of these plants in commercial rabbit production and to harness their full potential as tools for agroecological transition.

**Nomenclature**
ADF: Acid detergent fiber
ADL: Acid detergent lignin
ALT: Alanine transaminase
AST: Aspartate transaminase
AWG: Average weight
AWG: Average weight gain
CF: Crude fiber
CP: Crude protein
DM: Dry matter
EDTA: Ethylenediaminetetraacetic acid
AF: Abdominal fat
FCR: Feed conversion ratio
FECRT: Fecal egg count reduction test
HSD: Honestly significant difference
HPLC: High‐performance liquid chromatography
kcal/kg: Kilocalorie per kilogram
LANA: Laboratory of Food and Animal Nutrition
ME: Metabolizable energy
NDF: Neutral detergent fiber
NNE: Non‐nitrogenous extracts
OG: *Ocimum gratissimum*
OM: Organic matter
SEM: Standard error of the mean
VA: *Vernonia amygdalina*
%: Percentage
WIP: Water‐insoluble pectin

## Author Contributions

Basile Konmy: conceptualization, formal analysis, investigation, methodology, software, writing–original draft, and writing–review and editing; Christian C. Dansou: investigation, methodology, and writing–review and editing; Fiacre L. M. Acakpo Doumavo: writing–review and editing; Fallone B. Ganyé: investigation, resources, and writing–review and editing; Tony T. B. A. Sounkere: investigation, resources, and writing–review and editing; Rodrigue Towanou: investigation and writing–review and editing; Erick Virgile Bertrand Azando: conceptualization, resources, supervision, validation, writing–original draft, and writing–review and editing; Lamine Baba‐Moussa: resources, supervision, validation, visualization, writing–original draft, and writing–review and editing; Sanny‐Yo Doko Allou: conceptualization, funding acquisition, project administration, resources, supervision, validation, visualization, writing–original draft, and writing–review and editing; A. Pascal Olounladé: project administration, conceptualization, methodology, project administration, funding acquisition, resources, software, supervision, validation, visualization, writing–original draft, and writing–review and editing.

## Funding

No funding was obtained for this study.

## Conflicts of Interest

The authors declare no conflicts of interest.

## Supporting Information

Additional supporting information can be found online in the Supporting Information section.

## Supporting information


**Supporting Information 1** Supporting Information S1. Supporting Figure S1: Photographic documentation of experimental plants, including (a) *Vernonia amygdalina* whole plant, (b) *Vernonia amygdalina* leaves, (c) *Ocimum gratissimum* whole plant, and (d) *Ocimum gratissimum* leaves.

**Supporting Information 2** Supporting Information S2. Supporting Figure S2: Microscopic images illustrating helminth eggs and *Eimeria* spp. oocysts observed in rabbits fed *Ocimum gratissimum* and *Vernonia amygdalina* leaves.

**Supporting Information 3** Supporting Information S3. Supporting Figure S3: Q–Q plots and residuals versus fitted values used to assess model assumptions for zootechnical parameters of rabbits fed *Ocimum gratissimum* and *Vernonia amygdalina* leaves.

## Data Availability

The data that support the findings of this study are available from the corresponding author upon reasonable request.
