# Supplementary material for: Effects of Dietary Inclusion of Ocimum gratissimum and Vernonia amygdalina Leaf Meals on Growth Performance, Carcass Traits, Blood Profile, and Gastrointestinal Parasites in Weaner Rabbits
Source: Vet Med Int. 2026 Feb 12;2026:1803252. doi: 10.1155/vmi/1803252 (PMC12902444; doi:10.1155/vmi/1803252)
Supplement: Supplementary file 1 — Supporting Information 1 Supporting Information S1. Supporting Figure S1: Photographic documentation of experimental plants, including (a) Vernonia amygdalina whole plant, (b) Vernonia amygdalina leaves, (c) Ocimum gratissimum whole plant, and (d) Ocimum gratissimum leaves. [file VMI-2026-1803252-s003.pdf]

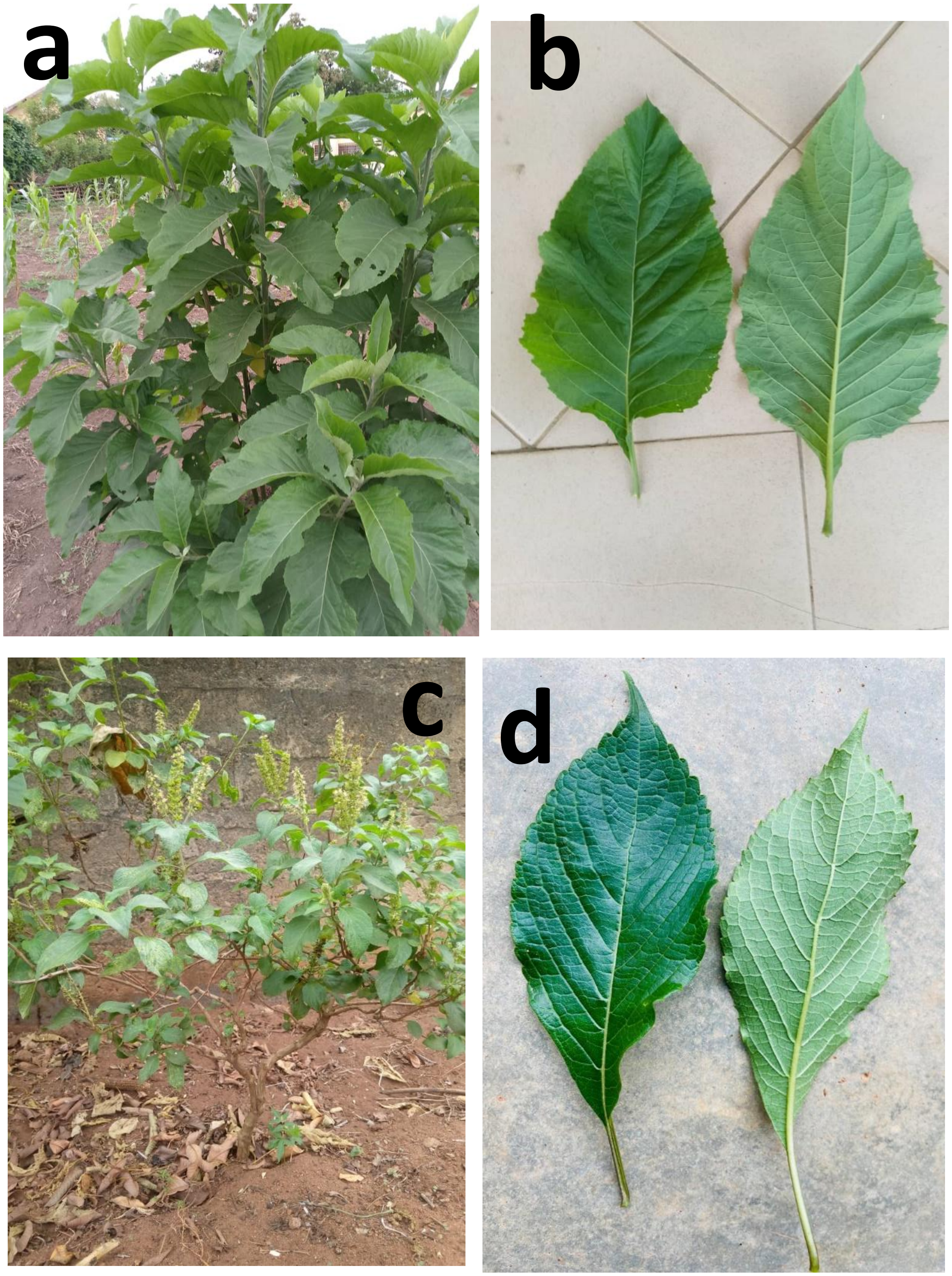

Supplementary Material **1** Supplementary Figure S 1: *Vernonia amygdalina* and *Ocimum gratissimum* plants.

(a)*Vernonia amygdalina* whole plant; (b) *Vernonia amygdalina* leaves; (c) *Ocimum gratissimum* whole plant; (d) *Ocimum gratissimum* leaves
