# Supplementary figures and images for: Effects of Dietary Inclusion of Ocimum gratissimum and Vernonia amygdalina Leaf Meals on Growth Performance, Carcass Traits, Blood Profile, and Gastrointestinal Parasites in Weaner Rabbits
Source: Vet Med Int. 2026 Feb 12;2026:1803252. doi: 10.1155/vmi/1803252 (PMC12902444; doi:10.1155/vmi/1803252)

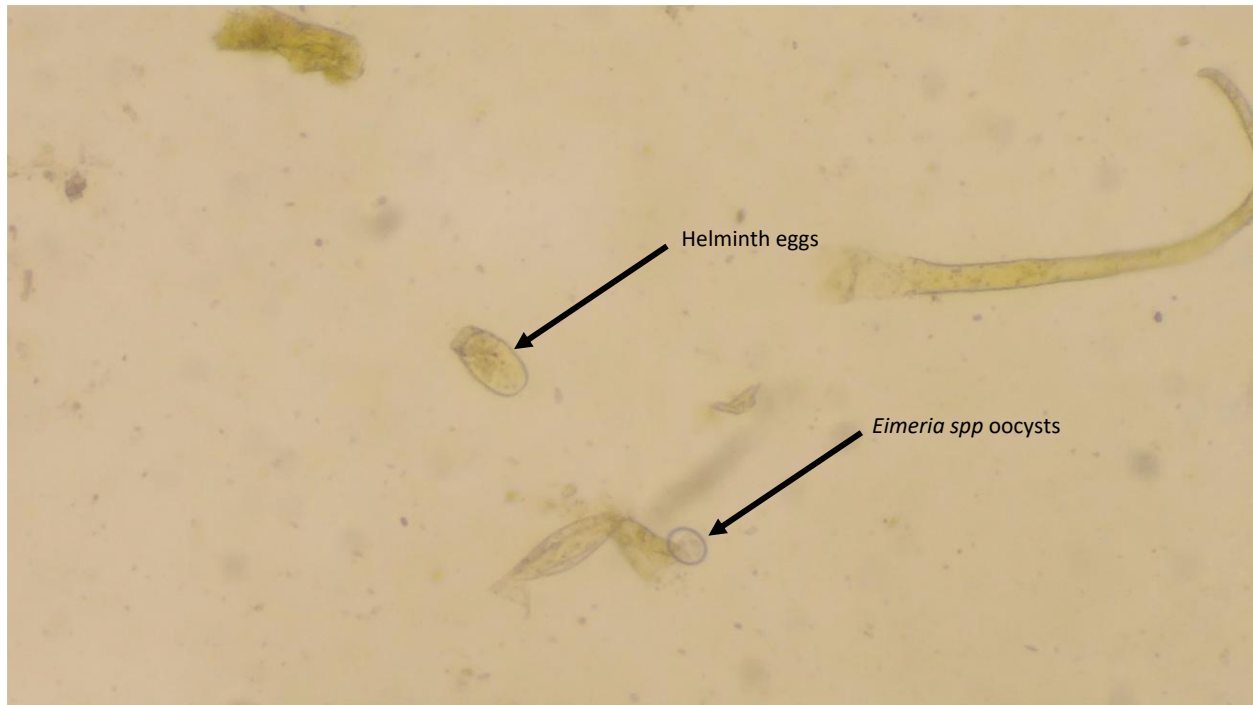

Supplementary Material 2 Supplementary Figure S 2: Helminth eggs and *Eimeria* Spp Occysts

Supplement: Supplementary file 2 — Supporting Information 2 Supporting Information S2. Supporting Figure S2: Microscopic images illustrating helminth eggs and Eimeria spp. oocysts observed in rabbits fed Ocimum gratissimum and Vernonia amygdalina leaves. [file VMI-2026-1803252-s001.pdf]
